# Macromolecules as targeted drugs delivery vehicles: an overview

**DOI:** 10.1080/15685551.2019.1591681

**Published:** 2019-04-05

**Authors:** Yousaf Ali, Ali Alqudah, Sadiq Ahmad, Shafida Abd Hamid, Umar Farooq

**Affiliations:** a Department of Chemistry, Sarhad University of Science and Information Technology, Peshawar, Pakistan; b Applied Biological Department, Tafila Technical University, Tafilah, Jordan; c Department of Pharmacy, University of Malakand, Malakand, Pakistan; d Department of Chemistry, Kulliyyah of Science, International Islamic University Malaysia, Kuantan, Malaysia; e Department of Pharmacy, Sarhad University of Science and Information Technology, Peshawar, Pakistan

**Keywords:** Macromolecules, drug delivery, polymer, drug-conjugate

## Abstract

Targeted drug delivery system improves the efficiency and safety of the therapeutic agents by managing the pharmacokinetics and pharmacodynamics of drugs. Currently, numerous drug carrier systems have been developed with different sizes, architectures and characteristics surface properties. Among different systems, macromolecules have a wide range of applications in targeted drug delivery system. The optimal drug loading potential, smooth drug releasing ability and biocompatibility are the distinguishing features that ensure the drugs delivery ability of macromolecules. This review briefly introduces some of the most commonly studied macromolecules which have been recommended as drugs delivery vehicles.

## Introduction

Targeted drug delivery system (TDDS) is a distinctive technique where the drug is administered to the desired location instead of the whole body or organ [1]. The selectivity improves the efficiency and safety of the therapeutic agents by controlling the rate, time, and location of an administrated drug. TDDS combines diverse fields of science like polymer science, pharmacology, bioconjugate chemistry and molecular biology. The objectives of the TDDS is to manage the pharmacokinetics, pharmacodynamics, non-specific toxicity, immunogenicity and bio-recognition of therapeutic agents [2].

**Figure 1.**
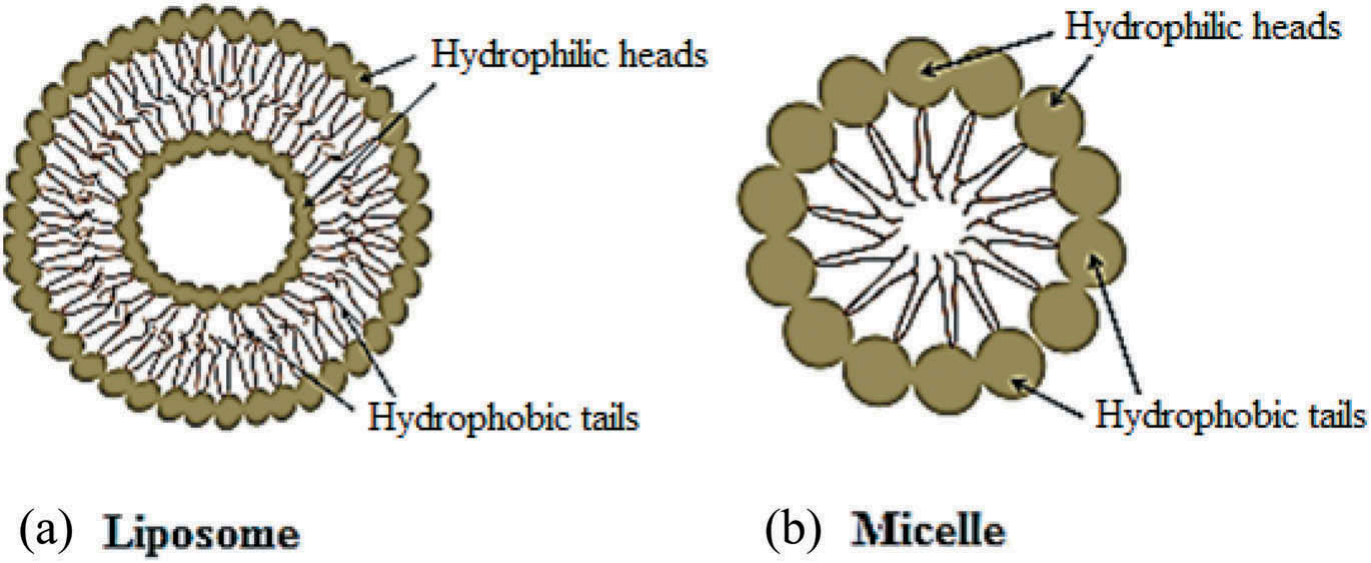
General representation of (a) liposome and (b) micelle.

**Figure 2.**
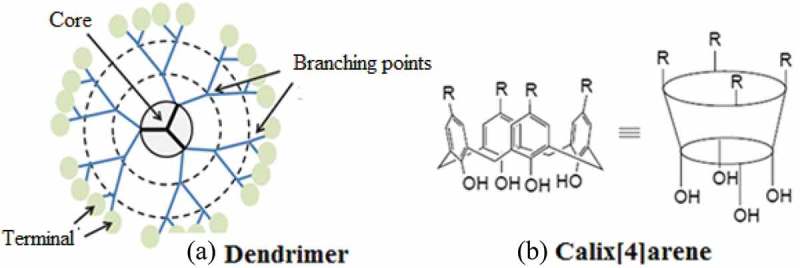
Structures of (a) dendrimer and (b) Calix[4]arene.

**Figure 3.**
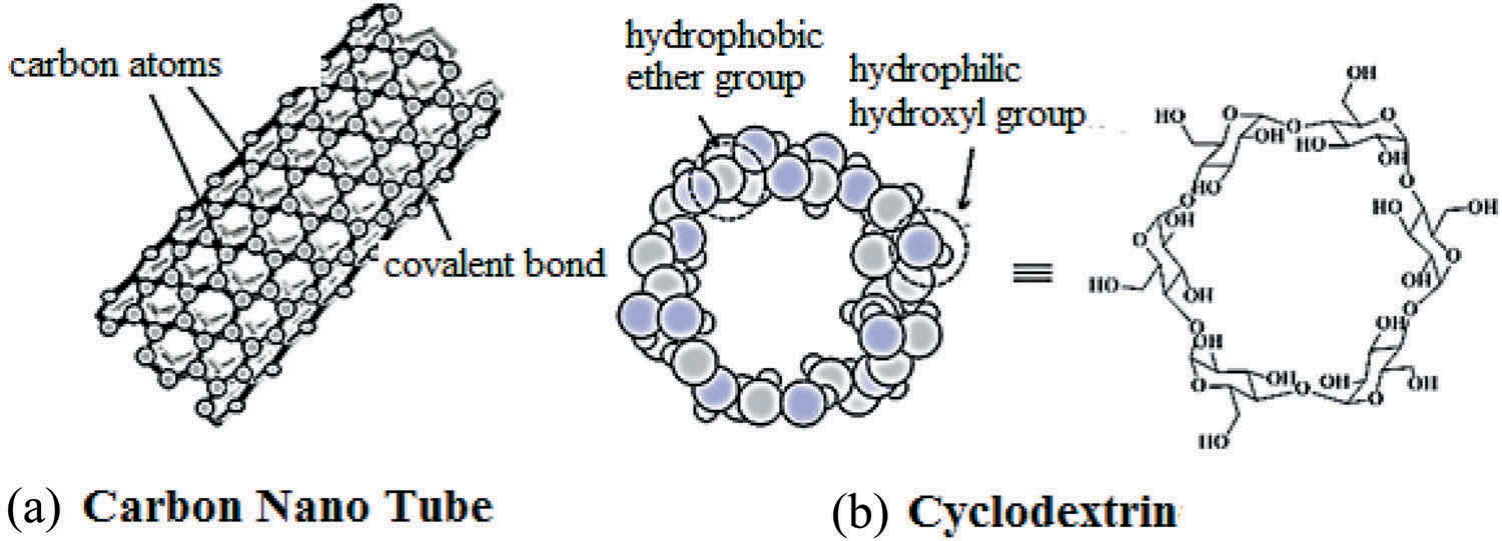
Representation of (a) carbon nano tube and (b) cyclodextrin.

The applications of TDDS are observed in various clinical conditions, including various types of cancer treatments. Chemotherapy has become an integral component of for most cancers treatment. Despite the last 30 years of effort on oncology drug discovery, conventional chemotherapeutic agents still exhibit poor specificity in reaching tumour tissues and are often restricted by dose-limiting toxicity. Concurrent and adjuvant/neo-adjuvant chemotherapies are usually practised along with the radiotherapy and surgery [3]. However, the administrated drugs not only target the cancer cells but also affect the normal healthy cells. The non-selectivity of cancer drugs is one of the main disadvantages that limit the effectiveness of chemotherapy. Thus to overcome these drawbacks of of conventional anti-cancer therapies, TDDS provides a suitable platform for the selective admiration of anticancer agents and thus decrease the harmful effects of treatment [4].

The concept of TDD dates back to 1906 when Paul Ehrlich (German Nobel laureate) proposed ‘magic bullet’ [5]. However, the field of TDDS emerged in the early 1970s.Currently, due to remarkable progress in materials science and pharmaceutics, several drug carriers have been prepared with different sizes, architectures and characteristics surface properties. These include conjugated polymers, polymeric micelles, liposomes, dendrimers, polymeric nanoparticles (NPs), viral NPs, and inorganic NPs made of iron oxide, quantum dots and gold NPs [6]. The common features required for anideal drug carrier system are optimal drug loading potential, long shelf life, provide smooth and sustained drugrelease having a good safety profiles [7]. The aim of this review isto present the drugs loading features of selected macromolecules, rather than an illustration of individual publications.

## Liposomes

Liposomes are among the commonly studied drug delivery systems (Figure 1a). The word liposome is derived from two Greek words; ‘Lipos’ which means fat and ‘soma’ means body [8]. Liposomes possess diverse range of morphologies and played a significant role in formulation of potent drug to improve therapeutics. Recently the liposome formulations are targeted to reduce toxicity and increase accumulation at the target site Current applications of liposomes are mainly observed in anticancer therapy; however, some liposomes have also been used for the treatment of other chronic diseases like Alzheimer [9].

The significant physical and chemical properties of liposomes are due to its main constituent, phospholipids [10]. The phospholipid bilayer of liposomes, which surrounds the internal aqueous core, is capable of drugs encapsulation. Hydrophilic and amphiphilic molecules are trapped in the core while hydrophobic molecules are partitioned into the lipid bilayer membrane. The encapsulation protects the drug molecules from degradation during systemic circulation as well as helpsin diminishing the side effects. For example, anti-tumour drug doxorubicin had been formulated as liposome in 1980s to improve the therapeutic index and decline the cardio-toxicity. Myocet and Doxil were the fist-approved liposome-based formulations for the treatment of carcinoma. Currently, several chemotherapeutic agents are in pre-clinical and clinical trials with auspicious results [4,11,12]. More than 40 liposome-based therapeutic agents are either inclinical use such as Doxil®, Ambisome®, and DepoDur™, or in various phases of clinical trials.

Functionalization of liposomes could be attained through various techniques. Among these, adsorption method is an easily approachable method where liposomal dispersion is incubated with the solution of targeting agent. However, the rapid desorption rate during storage and in vivo system make it less superior [8]. An alternate method is establishing covalent linkage between a ligand and liposome. The functional groups of targeted ligand, such as hydroxyl, carboxyl groups, are chemically attached to the appropriate component of liposomes. Besides, some drug molecules can also be attached to liposomes via suitable linkers. A linker covalently binds to the phospholipid head groups of liposome on one side and that to the targeted ligand at the other end [13].

To ensure the encapsulation of hydrophobic drugs into the inner hydrophilic core of liposome, new approaches have been developed that allow loading of hydrophobic substances in the inner aqueous part of liposome. Okamato et al., reported albumin-encapsulated liposome for the encapsulation of hydrophobic molecules (drugs). The modification was based on the assumption that attachment to albumin enhances aqueous solubility of hydrophobic agents. The size of bovine serum albumin(BSA)-liposomes can be regulated so as to target inflammatory sites and tumors via the EPR effect [14].

Various stimulants are used for the smooth detachment of therapeutic agents from the liposomes. The release of drug in the intended site is subjective to the membrane composition of the liposome and the nature of drugs attached to the liposomes [15]. The pH difference between normal and cancerous tissues is an environmental factor that facilitates the drug release from liposomes. pH-sensitive liposomes are suitable candidates for the release of drugs under acidic stimuli [16]. Similarly, the presence of certain enzymes provide reductive environment to the properly functionalized liposomes and release drug upon reduction. Besides, magnetic field, increased temperature and high-intensity focused ultrasounds are used as external stimuli trigger to release the drug [9].

## Micelles

Polymer micelles (PM) are promising vehicles that solubilise as well as carry/deliver poorly water-soluble therapeutic agents. Micelles are typically made up of 50–200 monomers. The diameter of PM ranges from 10–100 nm [17]. The size range of micelles provides stable long-term circulation in the main bloodstream. In addition, their small size has the advantage during the sterilisation processes in the pharmaceutical productions [18].

The micelles consist of an internal hydrophobic core and an external hydrophilic surface [19]. Drug molecules are entrapped physically in the hydrophobic core and thus escaping the need of functional groups for the encapsulation of drug molecules. The drug loading ability of micelles can be enhanced by their chemical conjugation to the amphiphilic polymers. This also prevents the premature release of the drug entities. The hydrophobic core boosts the transportation of those drugs that have low or no aqueous solubility, and therefore, polymeric micelles improve the therapeutic window of lipophilic anticancer agents, such as taxanes and platinates [20]. Such approaches reduce the threat of accumulation of a hydrophobic drug during the course of intravenous administration and decline the formation of embolism. Further, the micelle structure is greatly stabilised by the hydrophilic surface that decreases the chances of rapid clearance (Figure 1b). The prolonged circulation times in the *in vivo* system encourage its accumulation within the tumour cells [21,22].

The linkage of therapeutic agents to the surface of the micelles intensify cellular uptake in the cancer cells through receptor-mediated endocytosis. The first productive example of a tumour targeting polymeric micelle carrier was reported by Yokoyama et al. The doxorubicin entrapped polymeric micelle was found to be circulated in the bloodstream for long period and delivered to the tumour area at considerable higher concentrations compared tofree doxorubicin [13]. Various polymeric micelles are in clinical trail for investigation of their applications in targeted cancer therapy, solubilization of hydrophobic drugs and for the evasion of multidrug resistance. To date, one of them has been approved by FDA (Genexol™-PM) [23]. Drugs from the micellar systems can be easily released by internal or external triggered like pH, temperature or ultrasound.

## Dendrimers

Dendrimers are mono dispersed macromolecules with well-defined globular multi-branched structural units. They consist of three parts: a central focal point, repeated interior branching units, and multivalent exterior surface functional groups (Figure 2a). The polar drugs are trapped by electrostatical interaction while non-polar drugs are encapsulated within their hydrophobic fragment [24]. Dendrimers are mainly synthesised by two methods; a divergent method, where they grow outwards from a central core, and a convergent method in which the dendrimers are inwardly developed from the edge towards the central core. The branching units of dendrimers are designated in generation wise. The central branched core is named as Generation 0 (G0) and each successive addition of branching points are labelled as G1, G2, eand so on [25]. Most of their applications are due to their multifunctional structure with internal cavities. The uniform size distribution, globular design, high degree of branching units and functionalized surface are the characteristic features that render dendrimers special architecture to be used as drug delivery vehicles [26].

A drug is either non-covalently i.e. physically attached to its interior cavity, or covalently linked to the peripheral groups of the dendrimer. Gene plasmids and nucleic acids can be joined with dendrimers through electrostatic interaction. The covalent linkage is preferred as it provides a more stable platform for drug delivery. The discharge of the drug molecules depends on the nature of the dendrimers-drug attachment.

Dendrimers conjugated with transferrin or ligand specific for transferrin receptor have been reported for the treatment of cancer. Besides the drug pay load ability, dendrimers are appropriate components of molecular imaging contrast media [27]. DNA aptamer conjugated dendrimers have been recommended for imaging of cancer cells as well as for targeted cancer drug delivery. The encapsulated or chemically attached drug is then released by stimuli(pH, enzyme, etc.) [28].

## Calix[n]arenes

Calix[n]arenes are cyclic oligomers that are known as the 3^rd^ generation of supramolecular chemistry. They are generally synthesised from phenol and formaldehyde in an acidic or basic medium. The name was given due to the similarity in shape of one member of this class to a type of Greek vase, known as calix. The word ‘arene’ reflects their aromatic nature. The number of phenol groups varies from four to twenty [29]. A calixarene with four phenolic rings is generally symbolised as calix[4]arene (Figure 2b) while that with five aryl unit is symbolised as calix[5]arene and so on. Most commonly studied calix[n]arenes are that with n = 4, 6 and 8. Their cavity sizes are 3.0, 7.6 and 11.7 Å, respectively [30]. The Cup-shaped calix[n]arenes have the ability to accommodate drug-like molecules by forming inclusion complexes. Such accommodation in their cavities is an example of host-guest chemistry where calix[n]arene acts as a host and the drug condidate acts like a guest. The drug molecules are held by non-covalent interaction (hydrogen bonding and van der Waal’s) interactions. Inclusion complex of dinuclear platinum (anticancer drug) with *para-*sulfonato-calix[4]arene is an example of host-guest chemistry. In silico study of imatinib-calixarene complexes by Galindo-Mrillo suggested the controlled delivery of the drug (imatinib) into the cancer cells [3,31]. However, this area of research is still new and till date, no compound is in theclinicaltrailto confirm the validity of the existing in vivo and in silico studies.

## Carbon nanotubes

Carbon nanotubes (CNTs) were discovered in 1991 by Prof. Iijima [32]. These nano materials are made by rolling layers of graphene sheets (benzene rings) into seamless hollow cylinders, where the sp^2^-hybridized carbon atoms are organised hexagonaly (Figure 3a). Based on the number of graphene layers, these may be single-walled carbon nanotubes (SWNTs) or multi-walled carbon nanotubes (MWNTs) [33,34].The SWNTs havea diameter ranged from 0.4 to 2 nm and length between 20 and 100 nm, whereas, MWNTs are larger in size with a diameter ranged from 1.4 to 100 nm and length of 1 to several *µ*m [12]. The unique construction, light weight, high electrical and thermal conductivity, high mechanical strength, metallic/semi-metallic behaviour, expanded surface area and the ability to penetrate the membranes of different cells allowed them useful candidates in targeted drug delivery system [35]. The therapeutic agents are attached through covalent and non-covalentbinding, directed to the pathogenic site and released by different triggered mechanisms. Some nticancer drugs loaded in CNTs and conjugated with CNTs have shown promising results against various carcinomas. As an example, the activity of the platinum (IV) complex intensified over 100-fold when the drug was linked with the SWNTs [36].

## Cyclodextrin

Cyclodextrins (CDs) are cyclic oligosaccharides linked by α-1,4 glycosidic bonds which havetorus-like molecular shape with hydrophilic outer face and a lipophilic central cavity (Figure 3b). Most commonly studied CDs are α, β, and γ CDs, composed of six, seven and eight glucose units, respectively [37]. The hydrophobic cavity enables these macromolecules to form inclusion complexes with drug molecules and others. The drug-CD complex is stabilised by hydrogen bonding, van der Waals interactions and hydrophobic interactions. Like calixarenes, CDs were well known for host-guest chemistry and inclusion complexes. Along with this, CDs can accommodate drug molecules by non-inclusion manner. In the pharmaceutical industry, these are used for increasing aqueous solubility of drugs and their stability, masking odours and tastes of drugs, control release of solid dosage, and improving drugs bioavailability and permeability across biological barriers [38,39]. Currently, there are about 40 marketed pharmaceutical products based on CD complexes [40]. Some anticancer drugs complexed with cyclodextrin and their derivatives have shown improved physicochemical properties [41].

## Polymer-drug conjugates

Polymer-drug conjugates are among the commonly studied therapeutic carriers where a drug is covalently attached to the polymer, either directly or through a linker . There are three main components of a polymer-drug conjugate, a soluble polymer backbone, a biodegradable linker and covalently attached therapeutic agent(s) [42]. A linker is usually stimuli-responsive and controls the systematic releases of drug molecules under certain conditions. The polymer-drug conjugates system limits the cellular uptake to endocytosis and facilitates passive targeting of tumours through enhanced permeability and retention **(EPR)** effect. More so, Polymer-drug conjugates prolong the drug circulation times in the body, and protect the drug from premature degradation. Rings dorf (1975) introduced this concept and a few years later, Duncan & Kopecek designed the first targeted polymer-anticancer conjugates [43]. The area flourished in the subsequent years and many polymeric carriers have been reported, such as poly(vinyl alcohol), poly(vinyl pyrrolidone), polyglutamic acid and poly(malic acid). The two most applicableclasses are poly(ethylene glycol) (PEG)and *N*-(2-hydroxypropyl) methacrylamide (HPMA) copolymers [44].

The aqueous solubility, biocompatibility (the coexistence with living tissues without harm) and commercial availability are the characteristic features of PEG [45]. PEG possesses only two functional – OH groups for conjugation, however, it can be modified to obtain more sites for drug attachment. Branched, forked and multi-armed PEG systems are the examples of modified PEGs,which provide more space for drugs payload [46]. In linear PEG, drugs are only bind to the end of PEG chain, however, the modified PEGs have multiple hydroxyl groups for the drug attachment [47]. Doxorubicin, camptothecin, paclitaxel and palatinate drugs have been successfully conjugated to the functionalised PEG [48].

## 8. Azo polymer

Compounds containing at least one – N = N- functional group are called azo compounds. They have been reported as drug carriers by encapsulating drug molecules or by prodrug approach. In prodrug pathway, the therapeutic agents are conjugated via azo linkage and are subsequently released by the action of azo reductase enzyme. The azo reductase sensitive system is useful in colon-targeted drug delivery for the treatment of related diseases, such as colorectal cancer, inflammatory bowel disease and amoebiases [49]. Various polymer (liposomes, Micelles, PEG etc.) containing azo moiety are reported for targeted drug delivery. The drug is attached to the polymer via azo linkage (prodrug) and the polymer act as carrier moiety. Sometimes, the azo moiety just acts as a ‘gate keeper’’ that controls the release of drugs being loaded/attached to PEG, Micelles or liposomes. The encapsulated drugs are released in the desired region *by cis-trans* interchange of the azo part by an external stimulus (light or temperature). For example, Tong *et al*.synthesised amphiphilic diblock copolymer made of azo benzene polymethacrylate (PAzoMA) and poly(acrylic acid)(PAA) [50]. A reversible *trans-cis* photoisomerization was observed in the azo part by changing the UV/Vis light irradiation, that resulted in change in the morphology of polymer. Saint-Cricq *et al*. designed azo containing degradable nanoparticles for targeted drug delivery [51]. The nanoparticles were coated with the thermo-responsive azo-functionalised polymer (poly(ethylene glycol))(PEG).The model drug was released by the action of high frequency oscillating magnetic field.

## Conclusion

Targeted drug delivery system decreases the adverse effects of chemotherapy. Several drug carriers with different sizes, architectures and characteristics surface properties have been suggested for targeted drug delivery. Among these, macromolecules have been declared as potential candidates due to their optimal drug loading potential and smooth drug releasing ability. Drugs molecules can easily be attached to conjugated polymers, polymeric micelles, liposomes, dendrimers, polymeric nanoparticles due to their characteristics structural features. Stimuli-responsive azo-based compounds are useful in colon cancer and related diseases. Further consideration and interdisciplinary research might help to introduce a safer way of treatment, especially in the challenging field of cancer chemotherapy.
